# Prognostic value of inflammatory indices for atrial fibrillation recurrence after cryoablation: a cohort study

**DOI:** 10.3389/fcvm.2025.1637255

**Published:** 2025-09-17

**Authors:** Qiqiang Jie, Weichun Qian, Haibo Jia, Fengfu Zhang, Jianping Wang

**Affiliations:** ^1^ Department of Cardiology, Nanjing First Hospital, Nanjing Medical University, Nanjing, China; ^2^ Department of Geriatrics, Nanjing First Hospital, Nanjing Medical University, Nanjing, China

**Keywords:** atrial fibrillation, neutrophil-to-lymphocyte ratio, systemic immune-inflammation index, systemic inflammation response index, panimmune-inflammation value, cryoablation, recurrence

## Abstract

**Background:**

Inflammatory markers have emerged as potential prognostic markers of atrial fibrillation (AF) recurrence following cryoablation. However, comparative analyses of multiple systemic indices are limited. This study aimed to evaluate four inflammation-derived biomarkers—the neutrophil-to-lymphocyte ratio (NLR), systemic immune-inflammation index (SII), systemic inflammation response index (SIRI), and panimmune-inflammation value (PIV)—for their prognostic value in post-cryoablation AF recurrence.

**Methods:**

We conducted a retrospective cohort of 757 patients undergoing first-time cryoablation at Nanjing First Hospital (January 2017–December 2023). We investigated the associations between the four systemic inflammatory markers and AF recurrence. Baseline characteristics were collected from medical records, and inflammatory marker levels were calculated from routine blood tests. Multivariable Cox proportional hazards models were used to estimate adjusted hazard ratios; restricted cubic splines (RCS) assessed potential nonlinearity; and time-dependent receiver operating characteristic (ROC) analyses quantified predictive performance at 12 and 24 months.

**Results:**

Compared with tertile 1, tertile 3 showed higher multivariable-adjusted hazards of recurrence (HR: NLR = 4.112, SII = 4.010, SIRI = 5.137, PIV = 5.298; all *P* < 0.001). The RCS revealed inflection points (logNLR = 1.0, logSII = 6.0), beyond which the risk slopes intensified. Time-dependent ROC analyses showed the highest AUCs for logPIV (AUC = 0.764 at 12 months; 0.741 at 24 months) compared with the other indices (AUC range = 0.715–0.742), with an optimal cutoff yielding 79.2% sensitivity and 68.3% specificity.

**Conclusion:**

Systemic inflammation indices—particularly the pan-immune-inflammation value (PIV)—show prognostic association with AF recurrence after cryoablation and may inform preprocedural risk stratification and postablation surveillance. Given the observational design, these findings are associative and do not evaluate whether biomarker-guided selection or management improves outcomes. External calibration and validation—including in radiofrequency (RF) and pulsed-field ablation (PFA) cohorts—are needed to establish generalizability and clinical utility.

## 1 Introduction

Atrial fibrillation (AF), the most prevalent type of sustained cardiac arrhythmia, currently affects over 33 million individuals worldwide, with an increasing incidence linked to aging populations and cardiovascular comorbidities (1). While cryoablation has revolutionized rhythm control, with 70%–75% long-term success rates (2), substantial recurrence rates (25%–30%) underscore the urgent need for refined risk stratification tools. The 2024 ESC AF guideline acknowledges the role of inflammation—listing C-reactive protein among factors associated with incident AF and noting that biomarkers such as interleukin-6 may reflect residual stroke risk—yet it does not recommend routine biomarker-based stratification; consequently, clinically actionable inflammatory biomarkers beyond high-sensitivity CRP remain limited (3).

Accumulating evidence has implicatedhigher discriminative performance systemic inflammation in AF progression through multiple mechanisms: neutrophil extracellular traps (NETs) have been reported to promote fibrotic remodeling (4), platelet TGF-β1 release impairs conduction homogeneity (5), and lymphocyte‒macrophage crosstalk may sustain pro-inflammatory cytokine responses (6). Clinically, 40%–50% of AF patients demonstrate quantifiable inflammatory activation, with an elevated neutrophil‒lymphocyte ratio (NLR >3.0) being associated with higher postablation recurrence risk (7). By capturing multicellular inflammatory activity, composite indices—particularly the systemic immune-inflammation index (SII) and pan-immune-inflammation value (PIV)—have shown superior discrimination in some studies; for 1-year recurrence, PIV outperformed NLR (AUC = 0.768 vs. 0.691) (8, 9).

Despite these advances, several critical knowledge gaps remain: (1) most studies evaluate inflammatory markers in isolation, lacking direct comparisons of their predictive utility; (2) limited evidence exists regarding their specific prognostic value in cryoablation cohorts; and (3) optimal biomarker thresholds for clinical decision making remain undefined. To address these gaps, we conducted a large-scale retrospective cohort study (*n* = 757) of patients with AF undergoing cryoablation, comparing the prognostic value of four inflammation-based indices—neutrophil-to-lymphocyte ratio (NLR), SII, systemic inflammation response index (SIRI), and PIV. Our objectives were threefold: (1) to estimate clinically informative recurrence-associated thresholds, (2) compare prognostic (discriminative) performance across indices, and (3) identify the index with the most robust prognostic performance using time-dependent receiver operating characteristic (ROC) analyses.

## 2 Methods

### 2.1 Study population and design

**Figure 1:**
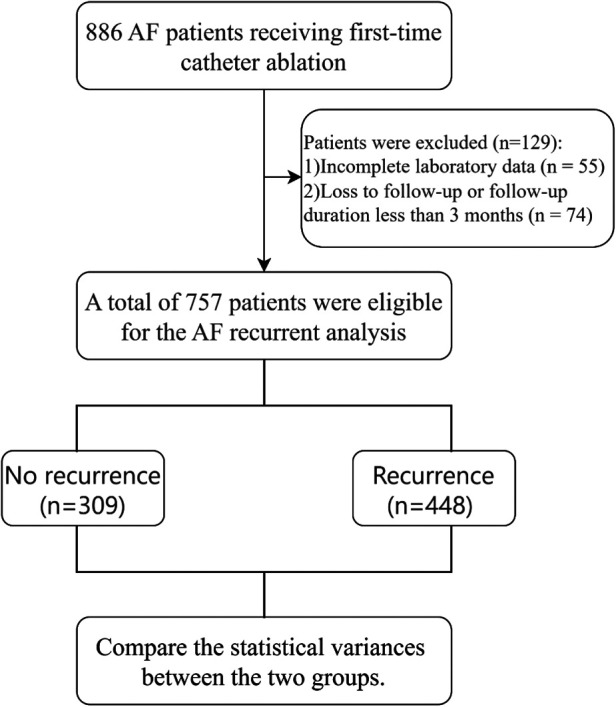
Flowchart of patient selection and data partitioning. A total of 886 atrial fibrillation (AF) patients who underwent first-time catheter ablation were initially screened. After 55 patients with incomplete echocardiographic or laboratory data and 74 patients who were lost to follow-up or had a follow-up duration of less than 3 months were excluded, a final study cohort of 757 patients was included.

This retrospective cohort study assessed 757 patients diagnosed with AF who underwent first-time cryoablation at the Cardiology Department of Nanjing First Hospital between January 2017 and December 2023. The diagnosis of AF was established on the basis of the following criteria: (1) documentation of AF on standard 12-lead ECG showing irregular RR intervals and no distinct P waves for at least 30 s; (2) 24-hour Holter monitoring confirming AF episodes; and (3) classification of the AF type (paroxysmal or persistent) on the basis of the 2020 ESC Guidelines for the diagnosis and management of AF. The eligibility criteria included a definitive diagnosis of AF via medical history, electrocardiogram (ECG), or Holter monitoring, coupled with a successfully executed cryoablation procedure using a second-generation (Arctic Front Advance, Medtronic, Inc., Minneapolis, MN, USA) cryoballoon catheter. The ablation strategy was standardized according to the type of atrial fibrillation: patients with paroxysmal AF underwent pulmonary vein isolation (PVI) only, whereas patients with persistent AF underwent PVI plus posterior wall isolation (PWI). The exclusion criteria were as follows: (1) previous AF ablation; (2) end-stage renal disease; (3) thrombus in the left atrium or its appendage; (4) valvular AF; (5) refusal or nonadherence to postoperative oral anticoagulant (OAC) therapy; (6) absence of blood cell count data; (7) acute inflammatory response, characterized by a white blood cell (WBC) count >10 × 10⁹/L or C-reactive protein (CRP) > 10 mg/L, or a diminished WBC count (<4 × 10⁹/L); and (8) autoimmune connective tissue disease or use of oral steroid medication. These criteria aimed to ensure a consistent study sample and reduce potential confounders that might compromise the reliability of the results. The study adhered to the principles outlined in the Declaration of Helsinki, and its protocol was approved by the Ethics Committee of Nanjing First Hospital. All patients provided written informed consent. Patients over the age of 75 were asked to sign a dual-signature informed consent form as an additional safeguard of their rights and to ensure that their family was involved in the decision-making process. Therefore, a patient and one relative (first degree) signed the informed consent form. This measure was not a substitute for patient consent, and no legal guardians signed on a patient's behalf. A flowchart of the participants is shown in Figure 1. Only cryoablation cases were included; no RF or PFA procedures were part of this cohort. Accordingly, generalizability to other ablation modalities cannot be assumed.

### 2.2 Data collection

Data collection was guided by biological considerations and the literature, ensuring that all potential prognostic variables were collected before ablation surgery. These variables included demographic and clinical characteristics such as age, sex, body mass index (BMI), type of atrial fibrillation (paroxysmal or persistent), duration of atrial fibrillation, and comorbidities such as smoking history, alcohol consumption history, hypertension history, cardiovascular disease (CVD) history, diabetes history, and prior stroke or transient ischemic attack (TIA). Risk stratification of patients was conducted via the CHA2DS2-VASc score. Blood samples collected within 24 h preceding the procedure were analyzed to determine the laboratory parameters. These included a complete blood count (comprising white blood cell count, neutrophil count, monocyte count, lymphocyte count, platelet count, red blood cell count, and hemoglobin level) and B-type natriuretic peptide (BNP) levels. BMI was calculated as weight (kg) divided by height (m) squared. Cardiac evaluation included recording standard 12-lead ECG parameters, such as the heart rate, PR interval, and QT interval, on the first postoperative day. Transesophageal echocardiography was conducted before ablation to determine the left atrial diameter, left ventricular ejection fraction (LVEF), and left atrial appendage flow velocity (LAAFV) while confirming the absence of intracardiac thrombi. The compiled data were meticulously reviewed by two clinicians, with any discrepancies subjected to in-depth evaluation by an electrophysiological medicine specialist within the cardiology department.

### 2.3 Assessment of systemic inflammatory indices

All blood samples were collected in the morning (between 7:00 AM and 9:00 AM) after an overnight fast and analyzed within 2 h of collection. The routine blood tests included white blood cell count (normal range: 4–10 × 10⁹/L), neutrophil count (2–7 × 10⁹/L), lymphocyte count (0.8–4 × 10⁹/L), monocyte count (0.12–0.8 × 10⁹/L), platelet count (100–300 × 10⁹/L), and hemoglobin level (130–175 g/L for males, 115–150 g/L for females). Several inflammatory markers are derived from these parameters: the NLR, which is calculated by dividing the absolute neutrophil count by the absolute lymphocyte count; the SII, which is calculated as the platelet count × neutrophil count/lymphocyte count; the SIRI, which is calculated as the neutrophil count × monocyte count/lymphocyte count; and the PIV, which is calculated as (neutrophil count × monocyte count × platelet count)/lymphocyte count. All indices were log-transformed (natural log) for modeling.

### 2.4 Follow-up protocol and outcome assessment

All patients were followed up at the outpatient clinic 1, 3, 6, and 12 months after ablation and every 6 months thereafter. The median follow-up duration was 24.5 months (interquartile range: 18.3–36.7 months). During each visit, the patients underwent 12-lead electrocardiography (ECG) and 24-hour Holter monitoring. Additionally, the patients were instructed to use a handheld ECG monitor (provided at discharge) to record their cardiac rhythm when experiencing symptoms suggestive of arrhythmia. AF recurrence was defined as any documented symptomatic or asymptomatic atrial tachyarrhythmia lasting ≥30 s after a 3-month blanking period. Time-to-event was measured from the end of the blanking period to the first documented recurrence or censoring at the last follow-up. Asymptomatic episodes were detected through routine Holter monitoring or occasional ECG examinations during the follow-up visits. For symptomatic episodes, documentation was obtained through handheld ECG monitoring, 12-lead ECG during unscheduled hospital visits, or 24-hour Holter monitoring. All documented arrhythmia episodes were independently reviewed by two electrophysiologists to ensure accurate classification, with any diagnostic discrepancies resolved by consensus.

### 2.5 Statistical analysis

Statistical analyses were conducted via SPSS 24.0 and R 4.0.2, with a significance threshold of *p* < 0.05. Variables with more than 30% missing values were removed, whereas those with fewer missing values were handled via multiple imputation by chained equations (MICE). Statistical tests were selected on the basis of data type and distribution: *t*-tests for normally distributed continuous variables, Mann‒Whitney *U*-tests for nonnormally distributed continuous variables, and chi‒square tests for categorical variables. Continuous variables are presented as the mean ± standard deviation or median (interquartile range) as appropriate.

The cutoff values for the NLR, SII, SIRI, and PIV were determined via ROC curve analysis to optimize sensitivity and specificity. These cutoffs were subsequently used to classify the markers into tertiles for further analysis. Multivariable Cox regression models were employed to assess associations with AF recurrence, adjusting for potential confounders, such as sex, age, BMI, and AF type. Comprehensive adjustments incorporated additional clinical and biochemical factors, including left atrial diameter, to ensure robust model performance. The results are presented as hazard ratios (HRs) with 95% confidence intervals (CIs).

Restricted cubic spline (RCS) analysis was used to explore the potential nonlinear relationships between AF recurrence and four systemic inflammatory markers. If the RCS analysis identified a distinct inflection point in the curve, indicating either a U-shaped or L-shaped pattern, the data were segmented into two independent parts on the basis of this inflection point for separate regression analysis. Additionally, the prognostic value of these inflammatory markers for 1-year and 2-year AF recurrence was evaluated via the area under the receiver operating characteristic (ROC) curve (AUC). For all the statistical tests, *p* < 0.05 (two-sided) was considered statistically significant.

## 3 Results

### 3.1 Comparison of baseline characteristics

A cohort of 757 patients with atrial fibrillation who underwent cryoablation was analyzed, comprising 448 patients with recurrence and 309 who remained recurrence-free (Figure 1). Table 1 presents the baseline characteristics, which showed significant between-group differences. Patients in the recurrence group presented a greater proportion of females (46% vs. 33%, *p* < 0.001), a greater prevalence of persistent atrial fibrillation (38% vs. 22%, *p* < 0.001), and elevated systolic blood pressure (133.7 ± 13.9 mmHg vs. 130.1 ± 13.0 mmHg, *p* < 0.001). No significant differences were observed between the two groups in terms of age, BMI, AF duration, CHA2DS2-VASc score, or underlying comorbidities (all *p* > 0.05). Patients without recurrence presented higher prescription rates of rivaroxaban (75% vs. 64%, *p* < 0.01), statins (41% vs. 25%, *p* < 0.001), and ARNIs (8% vs. 4%, *p* = 0.04). Significant variations were also observed in inflammatory markers and indices. Neutrophil levels were elevated in the recurrence group (4.7 ± 4.7 × 10⁹/L vs. 3.3 ± 1.6 × 10⁹/L, *p* < 0.001), whereas lymphocyte (1.8 ± 0.7 × 10⁹/L vs. 1.6 ± 0.7 × 10⁹/L, *p* < 0.001) and monocyte levels (0.6 ± 0.2 × 10⁹/L vs. 0.4 ± 0.1 × 10⁹/L, *p* < 0.001) were reduced. Inflammatory indices, including the NLR (3.7 ± 6.3 vs. 2.1 ± 1.4, *p* < 0.001), SII (645.5 ± 904.7 vs. 386.9 ± 294.2, *p* < 0.001), SIRI (2.0 ± 2.6 vs. 0.9 ± 0.7, *p* < 0.001) and PIV (349.6 ± 411.1 vs. 159.2 ± 137.4, *p* < 0.001), were significantly greater in the recurrence group. For log-transformed indices, the recurrence group presented significantly greater values for the logNLR (1.0 ± 0.6 vs. 0.6 ± 0.5, *p* < 0.001), logSII (6.2 ± 0.6, vs. 5.8 ± 0.6, *p* < 0.001), logSIRI (0.4 ± 0.7 vs. −0.4 ± 0.6, *p* < 0.001), and logPIV (5.6 ± 0.7 vs. 4.8 ± 0.7, *p* < 0.001). These comparisons support the prognostic relevance of systemic inflammatory activity for post-cryoablation AF recurrence.

**Table 1:** Baseline characteristics of 757 AF patients with and without recurrence after cryoablation.

Characteristic	Total (*n* = 757)	No recurrence (*n* = 309)	Recurrence (*n* = 448)	*P* value
Age (years)	60.2 ± 10.5	61.0 ± 9.9	59.5 ± 10.9	0.10
Female [*n* (%)]	306 (40)	102 (33)	204 (46)	<0.001
BMI (kg/m^2^)	25.1 ± 3.1	25.2 ± 3.1	25.0 ± 3.1	0.21
persistent AF [*n* (%)]	239 (32)	67 (22)	172 (38)	<0.001
AF History(months)	25.1 ± 42.7	23.6 ± 43.8	26.1 ± 41.8	0.13
CHA_2_DS_2_-VASc	1.7 ± 1.4	1.7 ± 1.5	1.6 ± 1.4	0.24
SBP (mmHg)	132.2 ± 13.7	130.1 ± 13.0	133.7 ± 13.9	<0.001
DBP (mmHg)	76.4 ± 14.2	76.9 ± 8.8	76.1 ± 16.9	0.02
Comorbidities [*n* (%)]				
Hypertension	390 (52)	161 (52)	229 (51)	0.85
Diabetes	107 (14)	47 (15)	60 (13)	0.55
Stroke/TIA	78 (10)	29 (9)	49 (11)	0.57
CAD	96 (13)	44 (14)	52 (12)	0.34
PCI [*n* (%)]	31 (4)	14 (5)	17 (4)	0.75
Hyperlipidemia	45 (6)	21 (7)	24 (5)	0.51
Smoking	157 (21)	66 (21)	91 (20)	0.80
Alcohol	121 (16)	43 (14)	78 (17)	0.23
Medications [*n* (%)]				
Propafenone	175 (23)	69 (22)	106 (24)	0.73
Amiodarone	287 (38)	123 (40)	164 (37)	0.41
Dronedarone	44 (6)	23 (7)	21 (5)	0.15
Wuxin particle	44 (6)	22 (7)	22 (5)	0.26
Beta-blockers	239 (32)	93 (30)	146 (33)	0.52
Rivaroxaban	521 (69)	233 (75)	288 (64)	<0.01
Dabigatran	212 (29)	80 (27)	132 (30)	0.40
Statins	239 (32)	127 (41)	112 (25)	<0.001
ARNIs	44 (6)	25 (8)	19 (4)	0.04
ACEI	50 (7)	23 (7)	27 (6)	0.53
ARBs	190 (25)	84 (27)	106 (24)	0.31
Loop diuretics	13 (2)	7 (2)	6 (1)	0.50
Thiazide diuretics	35 (5)	17 (6)	18 (4)	0.44
Spironolactone	22 (3)	9 (3)	13 (3)	1.00
Laboratory data				
Neutrophil, 10^9^/L	4.1 ± 3.8	3.3 ± 1.6	4.7 ± 4.7	<0.001
Lymphocyte, 10^9^/L	1.7 ± 0.7	1.8 ± 0.7	1.6 ± 0.7	<0.001
Monocyte, 10^9^/L	0.5 ± 0.2	0.4 ± 0.1	0.6 ± 0.2	<0.001
Platelet, 10^9^/L	187.3 ± 51.8	186.6 ± 50.8	187.8 ± 52.5	0.85
NLR	3.0 ± 5.0	2.1 ± 1.4	3.7 ± 6.3	<0.001
SII	539.9 ± 731.7	386.9 ± 294.2	645.5 ± 904.7	<0.001
SIRI	1.5 ± 2.1	0.9 ± 0.7	2.0 ± 2.6	<0.001
PIV	271.8 ± 341.1	159.2 ± 137.4	349.6 ± 411.1	<0.001
LogNLR	0.8 ± 0.6	0.6 ± 0.5	1.0 ± 0.6	<0.001
LogSII	6.0 ± 0.6	5.8 ± 0.6	6.2 ± 0.6	<0.001
LogSIRI	0.1 ± 0.8	−0.4 ± 0.6	0.4 ± 0.7	<0.001
LogPIV	5.3 ± 0.8	4.8 ± 0.7	5.6 ± 0.7	<0.001

AF, atrial fibrillation; ARBs, angiotensin II receptor blockers; ARNI, angiotensin receptor neprilysin Inhibitor; ACEI, angiotensin-converting enzyme inhibitor; BMI, body mass index; CAD, coronary artery disease; DBP, diastolic blood pressure; NLR, neutrophil to lymphocyte ratio; PCI, percutaneous coronary intervention; PIV, panimmune-inflammation value; SBP, systolic blood pressure; SII, systemic immune-inflammation index; SIRI, systemic inflammation response index; TIA, transient ischemic attack.

### 3.2 Systemic inflammation markers and AF recurrence

**Figure 2:**
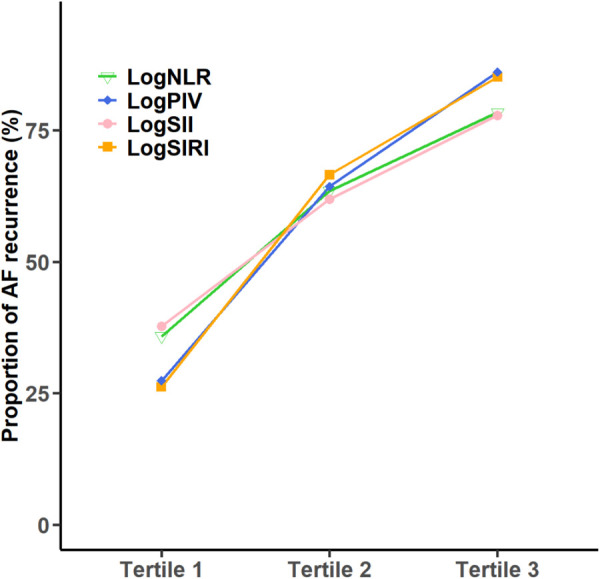
Af recurrence by tertiles of log-transformed inflammatory indices. Across all four indices (logNLR, logPIV, logSII, logSIRI), the proportion of AF recurrence increases from tertile 1 to tertile 3 (bars show percentages with 95% CIs; *P* for trend from the Cochran–Armitage test).

To further elucidate the relationship between systemic inflammation and the AF recurrence rate, we performed subgroup analyses by stratifying participants into three tertiles according to their log-transformed inflammatory indices (Figure 2). We analyzed the rate of AF recurrence in relation to the tertiles of the logNLR, logSII, logSIRI, and logPIV. For the logNLR, the recurrence rates in tertiles 1–3 were 91, 160, and 196 patients, representing 35.83%, 63.49%, and 78.40%, respectively. In the case of logSII, the recurrence rates for tertiles 1–3 were 95, 156, and 196 patients, with corresponding proportions of 37.70%, 61.90%, and 77.78%, respectively. For the logSIRI, the recurrence counts across tertiles 1–3 were 66, 169, and 212 patients, leading to percentages of 26.19%, 66.54%, and 85.14%, respectively. With respect to logPIV, tertiles 1–3 presented recurrence rates of 69, 162, and 217 patients, constituting proportions of 27.38%, 64.29%, and 86.11%, respectively. Taken together, these findings demonstrate a monotonic increase in recurrence across tertiles for all four indices.

### 3.3 Association between inflammatory markers and recurrence

Univariable Cox regression showed that higher neutrophil counts (HR = 1.083; 95% CI, 1.069–1.098; *p* < 0.001), NLR (HR = 1.056; 95% CI, 1.046–1.067; *p* < 0.001), SIRI (HR = 1.174; 95% CI, 1.148–1.200; *p* < 0.001), and PIV (per-unit HR = 1.001; 95% CI, 1.001–1.001; *p* < 0.001) were each associated with increased risk of atrial fibrillation recurrence (Table 2). Log-transformed indices exhibited larger effect sizes: logNLR (HR = 3.019; 95% CI, 2.588–3.522; *p* < 0.001) and logSII (HR = 3.225; 95% CI, 2.751–3.780; *p* < 0.001).

**Table 2:** Univariate Cox regression analysis of risk factors associated with atrial fibrillation recurrence.

Characteristic	HR (95% CI)	*P* value
Age (years)	1.001 [0.992–1.010]	0.799
Female [*n* (%)]	0.925 [0.767–1.114]	0.411
BMI (kg/m^2^)	0.981 [0.952–1.012]	0.23
persistent AF [*n* (%)]	1.082 [0.894–1.309]	0.419
AF History(months)	1.001 [0.999–1.003]	0.257
CHA_2_DS_2_-VASc	1.045 [0.978–1.116]	0.191
SBP (mmHg)	1.012 [1.006–1.018]	<0.001
DBP (mmHg)	1.006 [1.000–1.013]	0.065
Comorbidities [*n* (%)]		
Hypertension	1.167 [0.968–1.406]	0.106
Diabetes	0.997 [0.759–1.309]	0.982
Stroke/TIA	1.217 [0.904–1.639]	0.195
CAD	0.876 [0.656–1.170]	0.371
PCI [*n* (%)]	0.924 [0.569–1.501]	0.749
Hyperlipidemia	0.896 [0.594–1.352]	0.601
Smoking	0.869 [0.689–1.096]	0.236
Alcohol	0.941 [0.735–1.204]	0.626
Medications [*n* (%)]		
Propafenone	0.962 [0.772–1.199]	0.73
Amiodarone	1.124 [0.926–1.364]	0.236
Dronedarone	1.104 [0.711–1.714]	0.658
Wuxin particle	0.767 [0.628–0.936]	0.009
Beta-blockers	1.058 [0.870–1.286]	0.574
Rivaroxaban	0.872 [0.708–1.072]	0.194
Dabigatran	0.645 [0.520–0.800]	<0.001
Statins	0.962 [0.772–1.199]	0.73
ARNIs	1.145 [0.721–1.820]	0.57
ACEI	0.839 [0.568–1.239]	0.378
ARBs	0.924 [0.742–1.150]	0.48
Loop diuretics	0.987 [0.440–2.211]	0.974
Thiazide diuretics	0.925 [0.577–1.484]	0.75
Spironolactone	0.997 [0.573–1.733]	0.99
Laboratory data		
Neutrophil, 10^9^/L	1.083 [1.069–1.098]	<0.001
Lymphocyte, 10^9^/L	0.688 [0.591–0.801]	<0.001
Monocyte, 10^9^/L	1.240 [0.676–2.274]	0.488
Platelet, 10^9^/L	1.001 [0.999–1.003]	0.243
NLR	1.056 [1.046–1.067]	<0.001
SII	1.000 [1.000–1.001]	<0.001
SIRI	1.174 [1.148–1.200]	<0.001
PIV	1.001 [1.001–1.001]	<0.001
LogNLR	3.019 [2.588–3.522]	<0.001
LogSII	3.225 [2.751–3.780]	<0.001
LogSIRI	3.099 [2.685–3.577]	<0.001
LogPIV	3.247 [2.797–3.769]	<0.001

AF, atrial fibrillation; ARBs, angiotensin II receptor blockers; ARNI, angiotensin receptor neprilysin inhibitor; ACEI, angiotensin-converting enzyme inhibitor; BMI, body mass index; CAD, coronary artery disease; DBP, diastolic blood pressure; NLR, neutrophil to lymphocyte ratio; PCI, percutaneous coronary intervention; PIV, panimmune-inflammation value; SBP, systolic blood pressure; SII, systemic immune-inflammation index; SIRI, systemic inflammation response index; TIA, transient ischemic attack.

**Figure 3:**
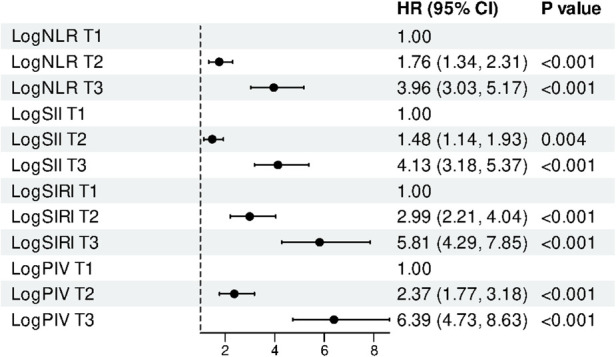
Multivariable-adjusted HRs (95% CI) for AF recurrence across tertiles of log-transformed inflammatory indices (logNLR, logPIV, logSII, logSIRI). Cox models adjusted for: Demographics (age, sex, BMI); AF/comorbidities (persistent AF, smoking, alcohol, hypertension, CAD, diabetes, NYHA class, CHA₂DS₂-VASc); Laboratory (TC, TG, HDL-C, LDL-C, BNP, hemoglobin, RBC, WBC, potassium, sodium, calcium); Medications (ARNI, rivaroxaban, statins); Echocardiography (left atrial diameter). T1 is reference; *P* for trend reported.

Multivariable Cox regression analysis revealed a significant association between higher tertiles of systemic inflammatory markers and an elevated risk of AF recurrence (Table 3). The associations for logNLR, logSII, logSIRI, and logPIV were statistically significant in the unadjusted, partially adjusted, and fully adjusted models. According to the fully adjusted model (Model 3), compared with individuals in the lowest tertile, those in the highest tertiles of the logNLR, logSII, logSIRI, and logPIV had 3.96-fold, 4.13-fold, 5.81-fold, and 6.39-fold higher adjusted hazards of AF recurrence, respectively (Table 3 and Figure 3).

**Table 3:** Associations between four systemic inflammatory markers and AF recurrence risk.

Variable	Model 1		Model 2		Model 3	
	HR (95% CI)	*P*	HR (95% CI)	*P*	HR (95% CI)	*P*
LogNLR categories						
T1	Reference		Reference		Reference	
T2	1.89 (1.46, 2.45)	<0.001	1.89 (1.46, 2.46)	<0.001	1.76 (1.34, 2.31)	<0.001
T3	3.91 (3.03, 5.04)	<0.001	3.93 (3.04, 5.07)	<0.001	3.96 (3.03, 5.17)	<0.001
*P* for trend	<0.001		<0.001		<0.001	
LogSII categories						
T1	Reference		Reference		Reference	
T2	1.55 (1.19, 2.00)	<0.001	1.56 (1.21, 2.02)	<0.001	1.48 (1.14, 1.93)	0.004
T3	3.98 (3.10, 5.11)	<0.001	4.08 (3.17, 5.25)	<0.001	4.13 (3.18, 5.37)	<0.001
*P* for trend	<0.001		<0.001		<0.001	
LogSIRI categories						
T1	Reference		Reference		Reference	
T2	2.69 (2.02, 3.58)	<0.001	2.75 (2.06, 3.67)	<0.001	2.99 (2.21, 4.04)	<0.001
T3	5.01 (3.79, 6.61)	<0.001	5.06 (3.83, 6.68)	<0.001	5.81 (4.29, 7.85)	<0.001
*P* for trend	<0.001		<0.001		<0.001	
LogPIV categories						
T1	Reference		Reference		Reference	
T2	2.18 (1.64, 2.89)	<0.001	2.26 (1.70, 3.01)	<0.001	2.37 (1.77, 3.18)	<0.001
T3	5.00 (3.80, 6.57)	<0.001	5.13 (3.89, 6.75)	<0.001	6.39 (4.73, 8.63)	<0.001
*P* for trend	<0.001		<0.001		<0.001	

HR, hazard ratio; CI, confidence interval. Model 1 was not adjusted for any confounders. Model 2 was adjusted for sex, age, type of atrial fibrillation, and body mass index. Model 3 was adjusted for age, body mass index, sex, persistent atrial fibrillation, smoking, alcohol consumption, hypertension, coronary artery disease, diabetes, New York Heart Association (NYHA) functional classification, CHA₂DS₂-VASc score, total cholesterol, triglycerides, high-density lipoprotein cholesterol, low-density lipoprotein cholesterol, B-type natriuretic peptide, angiotensin receptor-neprilysin inhibitors (ARNIs), rivaroxaban, statins, red blood cell count, white blood cell count, hemoglobin, potassium, sodium, calcium, and left atrial diameter.

**Figure 4:**
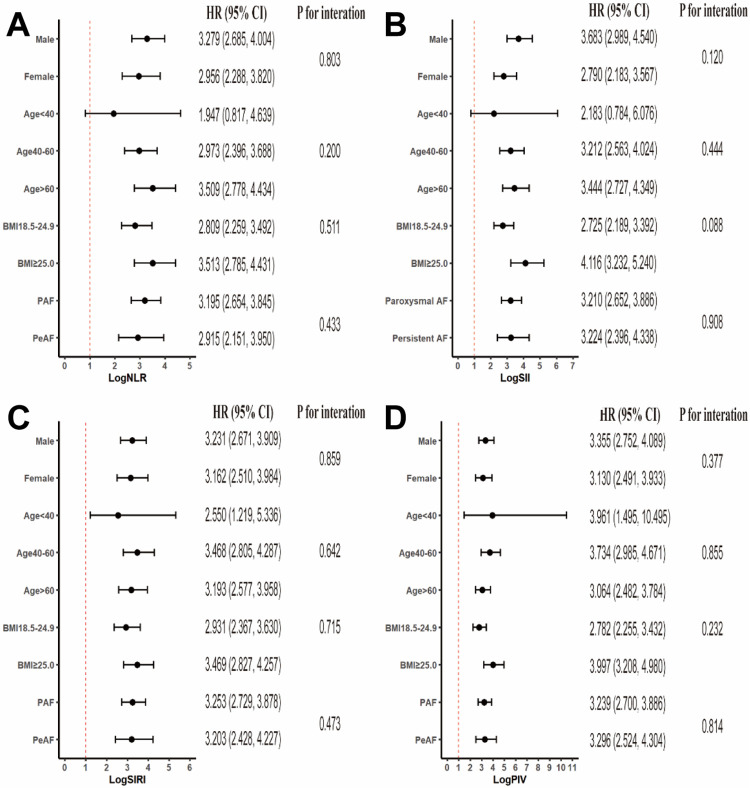
Subgroup analyses of the associations between systemic inflammatory markers and AF recurrence stratified by participant characteristics. The results are expressed as multivariable-adjusted HRs in continuous analyses after controlling for sex, age, atrial fibrillation type, body mass index, smoking, alcohol, hypertension, coronary heart disease, diabetes, NYHA functional classification, CHA2DS2VASc score, total cholesterol, triglycerides, low-density lipoprotein cholesterol, high-density lipoprotein cholesterol, glucose, glycated hemoglobin, and statins.

To assess the uniformity of the relationships between the four inflammatory indices (logNLR, logSII, logSIRI, and logPIV) and the recurrence of AF across diverse subgroups, we conducted a detailed subgroup analysis (Figure 4). This analysis included subgroups categorized on the basis of sex, age, BMI, and the nature of AF (paroxysmal or persistent). Relationships between all four inflammatory markers and AF recurrence were consistently observed across the subgroups, with no significant interactions for any variable (all *p* for interaction > 0.05). These findings indicate that the associations are consistent across demographic and clinical subgroups.

**Figure 5:**
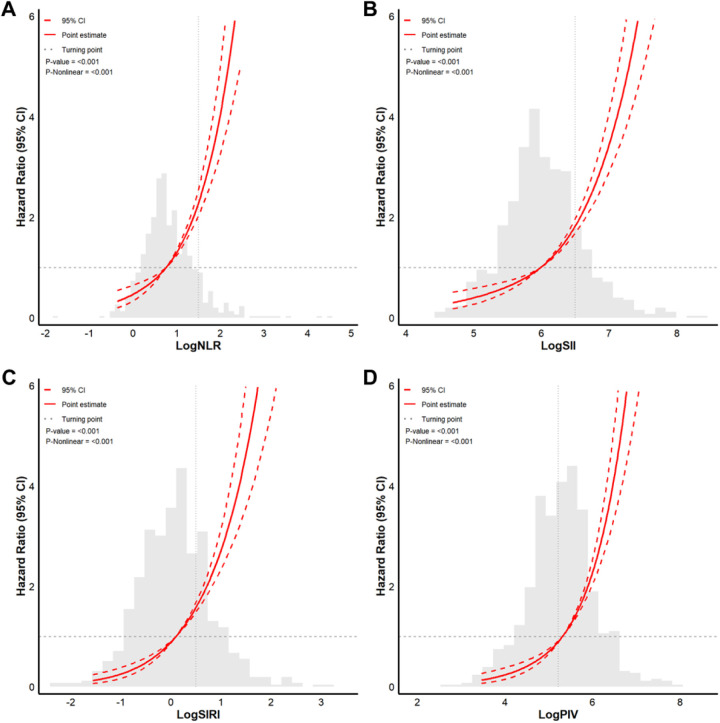
Restricted cubic spline (RCS) functions for the associations of logNLR, logSII, logSIRI, and logPIV with AF recurrence. Curves depict adjusted HRs (solid line) and 95% CIs (shaded bands). Vertical dashed lines mark data-driven inflection points used for segmented analyses.

RCS analysis revealed nonlinear associations of the logNLR, logSII, logSIRI, and logPIV with AF recurrence risk after adjustment for confounding factors (Figure 5). The inflection points were logNLR = 1.0, logSII = 6.0, logSIRI = 0.0, and logPIV = 5.0. According to the inflection points, we divided the data into two groups for each indicator (below or above the inflection point) and performed segmented regression analysis on the two groups (Table 4). When the logNLR was <1.0, a one-unit increase was associated with a 2.139-fold greater risk of AF recurrence (HR = 2.139, 95% CI: 1.372–3.336; *p* < 0.001). When the logNLR was greater than 1.0, a one-unit increase was associated with a 3.161-fold greater risk of AF recurrence (HR = 3.161, 95% CI: 2.362–4.229, *p* < 0.001). When the LogSII was lower than 6.0, a one-unit increase was associated with a 2.476-fold greater risk of AF recurrence (HR = 2.476, 95% CI, 1.470–4.172; *p* < 0.001). When the LogSII was greater than 6.0, a one-unit increase was associated with a 3.961-fold greater risk of AF recurrence (HR = 3.961, 95% CI: 3.138–4.999; *p* < 0.001). When the LogSIRI was lower than 0.0, a one-unit increase was associated with a 3.349-fold greater risk of AF recurrence (HR = 3.349, 95% CI, 1.693–6.627; *p* < 0.001). When the LogSIRI was equal to or greater than 0.0, a one-unit increase was associated with a 2.903-fold greater risk of AF recurrence (HR = 2.903, 95% CI: 2.320–3.633, *p* < 0.001). When logPIV was lower than 5.0, a one-unit increase was associated with a 4.366-fold greater risk of AF recurrence (HR = 4.366, 95% CI: 1.884–10.118, *p* < 0.001). When logPIV was ≥5.0, a one-unit increase was associated with a 3.487-fold greater risk of AF recurrence (HR = 3.487, 95% CI: 2.846–4.271; *p* < 0.001).

**Table 4:** Associations between four systemic inflammatory markers and AF recurrence risk.

Variable	HR	95% CI	*P*
LogNLR (<1.0)	2.139	(1.372, 3.336)	<0.001
LogNLR (≥1.0)	3.161	(2.362, 4.229)	<0.001
LogSII (<6.0)	2.476	(1.470, 4.172)	<0.001
LogSII (≥6.0)	3.961	(3.138, 4.999)	<0.001
LogSIRI (<0)	3.349	(1.693, 6.627)	<0.001
LogSIRI (≥0)	2.903	(2.320, 3.633)	<0.001
LogPIV (<5.0)	4.366	(1.884, 10.118)	<0.001
LogPIV (≥5.0)	3.487	(2.846, 4.271)	<0.001

### 3.4 Predictive performance of inflammatory indices for AF recurrence

**Figure 6:**
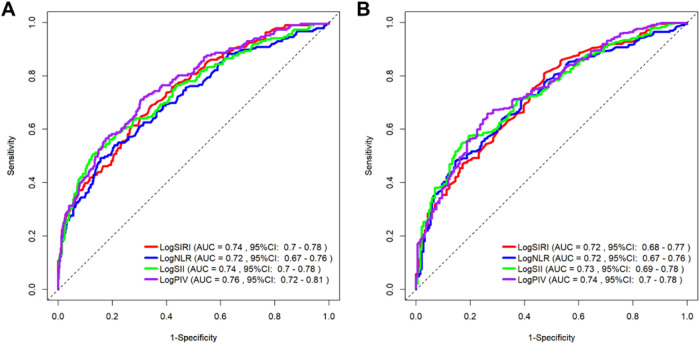
Receiver operating characteristic (ROC) curves of systemic inflammatory markers for predicting AF recurrence at one year **(A)** and two years **(B)**.

The time-dependent receiver operating characteristic (ROC) curves for evaluating the predictive efficacy of the logNLR, logSII, logSIRI, and logPIV for AF recurrence at 12 and 24 months are presented in Figures 6A,B. Tables 5, 6 show the corresponding areas under the curve (AUC) values and prognostic cutoff points. At the 12-month assessment, the AUC values were as follows: logNLR: 0.715 (95% CI: 0.669–0.761, *p* < 0.001), logSII: 0.740 (95% CI: 0.696–0.784, *p* < 0.001), logSIRI: 0.742 (95% CI: 0.699–0.784, *p* < 0.001), and logPIV: 0.764 (95% CI: 0.723–0.805, *p* < 0.001). The optimal prognostic thresholds for these markers were 0.644, 5.792, −0.005, and 5.132, respectively. The logPIV exhibited the highest sensitivity (79.2%) and specificity (68.3%), suggesting its superior predictive capacity during the initial year of follow-up. At the 24-month evaluation, the AUC values for the four indicators were as follows: logNLR: 0.719 (95% CI: 0.674–0.764, *p* < 0.001), logSII: 0.732 (95% CI: 0.688–0.776, *p* < 0.001), logSIRI: 0.724 (95% CI: 0.679–0.769, *p* < 0.001), and logPIV: 0.741 (95% CI: 0.698–0.785, *p* < 0.001). The cutoff values for these biomarkers in predicting AF recurrence remained consistent with those observed at 12 months. Among the four biomarkers, logPIV maintained the highest sensitivity (79.2%) and specificity (68.3%), as indicated in Table 5. In summary, the analysis revealed that the logPIV consistently demonstrated superior predictive capability for AF recurrence, whereas the logSII, logNLR, and logSIRI exhibited moderate prognostic efficacy throughout both study phases.

**Table 5:** Abilities of the LogNLR, LogSII, LogSIRI and LogPIV to predict AF recurrence at 12 months.

Variable	AUC	*P* value	95% CI	Se (%)	Sp (%)	PPV	NPV	Cutoff
LogNLR	0.715	*P* < 0.001	0.669–0.761	73.7	59.2	72.4	60.8	0.644
LogSII	0.74	*P* < 0.001	0.696–0.784	78.1	52.1	70.3	62.2	5.792
LogSIRI	0.742	*P* < 0.001	0.699–0.784	76.1	74.4	81.2	68.2	−0.005
LogPIV	0.764	*P* < 0.001	0.723–0.805	79.2	68.3	78.4	69.4	5.132

**Table 6:** Abilities of the LogNLR, LogSII, LogSIRI and LogPIV to predict AF recurrence at 24 months.

Variable	AUC	*P* value	95% CI	Se (%)	Sp (%)	Cutoff
LogNLR	0.719	*P* < 0.001	0.674–0.764	73.7	59.2	0.644
LogSII	0.732	*P* < 0.001	0.688–0.776	78.1	52.1	5.792
LogSIRI	0.724	*P* < 0.001	0.679–0.769	76.1	74.4	−0.005
LogPIV	0.741	*P* < 0.001	0.698–0.785	79.2	68.3	5.132

## 4 Discussion

This study assessed the associations between inflammatory indices (NLR, SII, SIRI, and PIV) and AF recurrence following cryoablation. Elevated preprocedural levels of these markers were significantly linked to an increased risk of recurrence. ROC analysis revealed strong predictive performance, with PIV exhibiting the highest accuracy (AUC = 0.764). A major strength of this study is the determination of clinically actionable thresholds, particularly for LogPIV. The optimal LogPIV cutoff at 12 months was 5.132 (sensitivity: 79.2%; specificity: 68.3%; PPV: 78.4%; NPV: 69.4%), providing clear clinical guidance. These findings suggest that preprocedural LogPIV can effectively aid patient counseling and identify patients who might benefit from intensified monitoring or adjunctive anti-inflammatory therapies postablation.

Our findings should be interpreted within the broader context of AF recurrence prediction. Recently, significant progress has been made in imaging-based risk assessment, particularly through evaluating left atrial structural remodeling (10). Techniques such as speckle-tracking echocardiography for measuring LA strain and late gadolinium enhancement cardiac MRI (LGE-MRI) for quantifying atrial fibrosis have provided valuable insights into the structural arrhythmogenic substrate (11, 12). However, these markers primarily reflect structural disease consequences (13). The inflammatory indices examined in our study add complementary value by quantifying systemic biological activity potentially driving substrate progression (14). Indeed, inflammation is increasingly recognized as a central factor in “atrial cardiomyopathy,” a concept encompassing the structural, electrical, and functional atrial abnormalities that predispose individuals to AF (15). Thus, integrating these readily available, cost-effective inflammatory biomarkers with advanced imaging techniques could significantly enhance future risk stratification models.

Previous studies have revealed the predictive capacity of individual inflammatory markers. However, our study supplemented the value of these biomarkers from a more integral perspective. For example, a high NLR was reported to predict a high risk of AF recurrence after ablation (16). Ding et al. reported that a higher preablation NLR was related to increased AF recurrence, which was in line with our results (17). Guo et al. reported that the postablation NLR can predict long-term AF recurrence, which further confirmed the significance of the NLR for predicting inflammation-related AF management (18). Kaplan et al. reported that the SII had a sensitivity of 71% in predicting AF recurrence in a European population (*n* = 370) (19), whereas Gu et al. reported a similar PIV predictive capacity (AUC = 0.768) in a Chinese population (*n* = 307) (9). In the present study, we obtained similar findings and expanded their scope of significance. The multibiomarker analysis strategy used in this study balanced the advantages of individual biomarkers and provided more information on their interrelationships and relative values. Furthermore, the 24-month follow-up duration in the current study filled the gap in knowledge of the long-term prognostic value of these biomarkers, which was a major limitation of most previous studies. Moreover, consistent findings across different population characteristics and follow-up durations strengthened the generalizability of these inflammatory markers.

The link between inflammatory markers and AF recurrence may be explained by several interrelated mechanisms. At the cellular level, neutrophils are essential for inflammation (20), fibrosis development, and atrial structural remodeling through the release of proinflammatory cytokines (21), reactive oxygen species, and matrix metalloproteinases (22). Lymphocytes participate significantly in the inflammatory response via numerous pathways, such as inflammatory cytokine secretion (23), immune cell mobilization (24), and direct interaction with cardiac cells (25). Platelets have nonhemostatic functions, including TGF-β1 secretion, fibroblast differentiation, and atrial fibrosis promotion (26, 27). These cellular interactions trigger and sustain a complex inflammatory cascade primarily mediated by key cytokines. Tumor necrosis factor-alpha (TNF-α) and interleukin-6 (IL-6) are major inflammatory mediators strongly associated with AF progression (28–30). These cytokines, along with C-reactive protein (CRP), activate a cascade of cellular events leading to fibrosis and collagen deposition in the atrial myocardium (31). The levels of additional proinflammatory cytokines, including interleukin-1 beta (IL-1β) (32) and monocyte chemoattractant protein-1 (MCP-1) (33), are significantly elevated in patients with recurrent AF. The intricate relationship between these cellular elements generates a proinflammatory state that may predispose patients to AF recurrence.

The strengths of our study are its baseline characteristics, statistical methodology, nonlinear models, and large sample size. We measured multiple inflammatory markers, allowing us to better evaluate the overall inflammatory status and its association with AF recurrence. Moreover, we used multivariable analysis and ROC curve analysis, which made our results more valid. The large sample size and long-term follow-up period also made our results more statistically significant and clinically relevant. Several limitations should be noted. First, this was a single-center observational cohort; causal inference is not possible, and residual or unmeasured confounding (e.g., medication adherence, lifestyle factors) may persist. Second, all patients underwent cryoablation—no radiofrequency (RF) or pulsed-field ablation (PFA) procedures were included—so generalizability to RF- or PFA-treated populations is uncertain and will require dedicated validation. Third, laboratory indices were derived from routine testing at a single institution; inter-laboratory differences in assay platforms and reference ranges may limit transportability. Fourth, inflammatory markers were measured once preprocedurally; we did not evaluate longitudinal dynamics, precluding analyses of time-varying associations. Fifth, ROC-derived cutoffs and the associated PPV/NPV are cohort-specific and require external calibration and validation before clinical use. Accordingly, any suggestion that these markers could optimize patient selection or management remains speculative and warrants prospective evaluation.

Future research should prioritize validation studies across diverse populations, longitudinal evaluations of marker fluctuations, interventional studies focused on inflammation, integration of markers into risk stratification models, and exploration of treatment impacts on inflammatory markers. In conclusion, our study presents compelling evidence supporting the predictive utility of inflammatory markers in predicting AF recurrence following cryoablation. These findings imply that the inclusion of these markers in clinical routines could enhance patient risk stratification and inform personalized treatment approaches. The thorough assessment of various inflammatory indices provides a sophisticated comprehension of the inflammatory mechanisms underlying AF recurrence, potentially leading to superior patient outcomes via enhanced risk prediction and management strategies.

## 5 Conclusion

In conclusion, elevated preprocedural NLR, SII, SIRI, and PIV were associated with AF recurrence after cryoablation. These inflammation-based indices may aid preprocedural risk stratification and tailored follow-up by helping identify higher-risk patients. However, the observational design precludes causal inference. Before clinical use, the proposed cutoffs and their prognostic utility require external calibration and validation—ideally with assessment of incremental value over established clinical predictors. Whether biomarker-guided management or anti-inflammatory interventions improve outcomes remains to be determined. External validation in RF and PFA cohorts is also needed to establish generalizability beyond cryoablation.

## Data Availability

The raw data supporting the conclusions of this article will be made available by the authors, without undue reservation.
